# Elucidating the role of maternal environmental exposures on offspring health and disease using two-sample Mendelian randomization

**DOI:** 10.1093/ije/dyz019

**Published:** 2019-02-27

**Authors:** David M Evans, Gunn-Helen Moen, Liang-Dar Hwang, Debbie A Lawlor, Nicole M Warrington

**Affiliations:** 1 University of Queensland Diamantina Institute, Translational Research Institute, Brisbane, Queensland, Australia; 2 Medical Research Council Integrative Epidemiology Unit, University of Bristol, Bristol, UK; 3 Population Health Sciences, Bristol Medical School, University of Bristol, Bristol, UK; 4 Department of Endocrinology, Morbid Obesity and Preventive Medicine, Oslo University Hospital, Oslo, Norway; 5 Institute of Clinical Medicine, Faculty of Medicine, University of Oslo, Oslo, Norway; 6 Bristol NIHR Biomedical Research Centre, Bristol, UK

**Keywords:** Maternal effects, fetal effects, offspring genetic effects, Mendelian randomization, DOHaD, Developmental Origins of Health and Disease, Fetal Insulin Hypothesis, birthweight, type 2 diabetes

## Abstract

**Background:**

There is considerable interest in estimating the causal effect of a range of maternal environmental exposures on offspring health-related outcomes. Previous attempts to do this using Mendelian randomization methodologies have been hampered by the paucity of epidemiological cohorts with large numbers of genotyped mother–offspring pairs.

**Methods:**

We describe a new statistical model that we have created which can be used to estimate the effect of maternal genotypes on offspring outcomes conditional on offspring genotype, using both individual-level and summary-results data, even when the extent of sample overlap is unknown.

**Results:**

We describe how the estimates obtained from our method can subsequently be used in large-scale two-sample Mendelian randomization studies to investigate the causal effect of maternal environmental exposures on offspring outcomes. This includes studies that aim to assess the causal effect of *in utero* exposures related to fetal growth restriction on future risk of disease in offspring. We illustrate our framework using examples related to offspring birthweight and cardiometabolic disease, although the general principles we espouse are relevant for many other offspring phenotypes.

**Conclusions:**

We advocate for the establishment of large-scale international genetics consortia that are focused on the identification of maternal genetic effects and committed to the public sharing of genome-wide summary-results data from such efforts. This information will facilitate the application of powerful two-sample Mendelian randomization studies of maternal exposures and offspring outcomes.

**Key Messages**

- Statistical methods exist for partitioning genetic effects at single loci into maternal and offspring genetic components.
- Estimates of maternal genetic effects obtained from these methods can be used in large-scale two-sample Mendelian randomization studies to investigate the causal effect of maternal environmental exposures on offspring outcomes.
- Two-sample Mendelian randomization studies can also be used to assess the causal effect of *in utero* exposures related to fetal growth on future risk of disease in offspring.

## Introduction

There is considerable interest in elucidating the causal effect of maternal environmental exposures on offspring outcomes. However, traditional observational epidemiological studies are prone to confounding, bias and reverse causality. Mendelian randomization (MR) is an epidemiological method that was developed to estimate the causal effect of environmental exposures on medically relevant outcomes.^1^ Recently, several studies have attempted to use MR to investigate the causal effect of maternal environmental exposures on offspring outcomes.^2^^,^^3^ However, most of these studies have been performed in relatively small samples of genotyped mother–offspring pairs because offspring genotypes are needed to prevent violation of the assumptions underlying MR. This has consequently limited the statistical power, utility and broader application of the MR method in investigating the causal effect of maternal environmental exposures on offspring outcomes.

In this manuscript, we describe a statistical method based on structural equation modelling that we have recently developed to partition genetic effects on offspring phenotypes into maternal and offspring components.^4^ We discuss how this partitioning could be used to facilitate large-scale two-sample MR studies of maternal exposures and offspring outcomes in different samples of individuals, maximizing sample size and obviating the requirement of individual-level genotyped mother–offspring pairs. Within this context, we show how the recent identification of genetic loci that exert maternal effects on offspring birthweight^5^ provides an opportunity to assess the causal effect of *in utero* exposures related to fetal growth on offspring outcomes using this method. We illustrate our methods using an example involving susceptibility to type 2 diabetes and offspring birthweight. Our methods have broad applicability to investigating hypotheses involving the Developmental Origins of Health and Disease (DOHaD).^6^ Finally, we advocate for the establishment of large-scale genetics consortia whose remit is to identify maternal genetic effects and utilize this information to elucidate the role of maternal environmental factors on offspring outcomes via MR using the methods that we espouse in this manuscript.

## Partitioning genetic effects at individual loci into maternal and offspring components

The resolution of genetic effects into maternal and offspring components is important because it not only provides meaningful insights into the mechanism through which genotypes exert their effects on offspring phenotypes, but it can also be leveraged for a number of informative downstream analyses including MR.^1^^,^^4^^,^^7^ Traditionally, in human genetic association studies, the estimation of maternal genetic effects on offspring phenotypes has been achieved through conditional genetic association analysis of genotyped mother–offspring pairs. For example, one regresses offspring phenotype on maternal genotype whilst controlling for the possible confounding influences of the offspring’s genotype:

(1) $$Y_{i}=\alpha +\beta_{o}\times \mathrm{oSN}P_{i}+\beta_{m}\times \mathrm{mSN}P_{i}+\varepsilon_{i}$$

where *Y_i_* and *oSNP_i_* refer to the (offspring) phenotype and SNP genotype of the *i*th individual, *mSNP_i_* is the genotype of the individual’s mother at the same locus, *β_o_* and *β_m_* the estimated offspring and maternal effects of the SNP, *α* is an intercept and *ε_i_* a normally distributed residual term. However, a problem with this simple approach is that there is a paucity of cohorts worldwide with genome-wide association study (GWAS) data on both mothers and their offspring. Additionally, loci influencing complex traits in the offspring are typically of small effect and, since maternal and offspring genotypes are highly correlated, power is often low to definitively partition genetic effects into maternal and offspring components. For example, in a recent study of offspring birthweight, we initially attempted to partition the genetic effect at 58 autosomal genome-wide significant birthweight SNPs into maternal and offspring components using simple conditional linear regression in some of the largest genotyped cohorts of mother–offspring pairs that were available at the time (total *N* = 12 909). However, we were unable to resolve these effects definitively at nearly all of the birthweight loci.^8^

**Figure 1.**
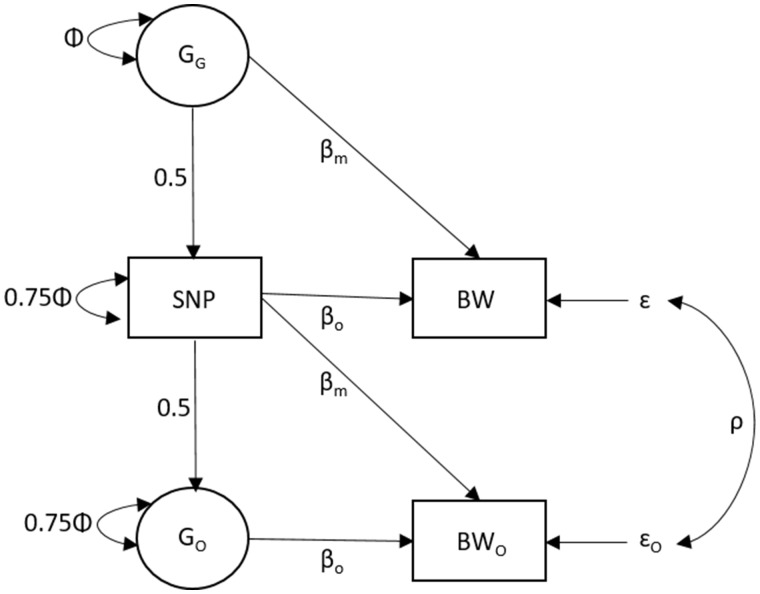
Structural equation model (SEM) used to estimate maternal and offspring genetic effects on birthweight. The three observed variables (in squares) denote the birthweight of a UK Biobank individual (BW), the birthweight of their offspring (BW_O_) and their own genotype (SNP). The latent unobserved variables (in circles) represent the genotypes of the individual’s mother (their offspring’s grandmother; G_G_) and the genotype of the individual’s first offspring (G_O_). The total variance of the latent genotypes for the individual’s mother (G_G_) and offspring (G_O_) and for the observed SNP variable are set to Φ and are estimated from the data. The causal path between the individual’s own genotype and both their mother and offspring’s latent genotype is set to 0.5. The *β_m_* and *βo* path coefficients refer to maternal and offspring genetic effects on birthweight, respectively. The residual error terms for the birthweight of the individual and their offspring are represented by *ɛ* and *ɛ_O_*, respectively, and the variance of both these terms is estimated in the structural equation model. The covariance between the error terms is denoted by *ρ*. The model can be modified easily to include observed genotypes and/or the absence of one of the birthweight phenotypes.

Recently, we developed a new statistical model based on the mathematical technique of structural equation modelling, which has allowed us to estimate maternal and offspring effects on birthweight utilizing data from the large UK Biobank Study.^4^^,^^9^ A key realization was that the UK Biobank Study contains self-reported information not only on individuals’ own birthweight, but also (in the case of females) on the birthweight of their first-born offspring, and this makes it possible to resolve both maternal and offspring effects on birthweight in the cohort. Figure 1 illustrates the mathematical features of our structural equation model (SEM) in the form of a path diagram. One-headed arrows represent causal paths and two-headed arrows correlational relationships. The three observed variables (in squares) denote the birthweight of a UK Biobank individual (BW), the birthweight of their offspring (BW_O_) and their own genotype (SNP). The latent unobserved variables (in circles) represent the genotypes of the individual’s mother (their offspring’s grandmother; G_G_) and the genotype of the individual’s first-born offspring (G_O_). The total variance of the latent genotypes for the individual’s mother (G_G_) and offspring (G_O_) and for the observed SNP variable are all set to Φ [i.e. variance(G_G_) = Φ, variance(SNP) = 0.75Φ + 0.25Φ, variance(G_O_) = 0.75Φ + 0.25Φ, as can be verified by path analysis/covariance algebra], which is estimated from the data. According to quantitative genetics theory, the causal path between the individual’s own genotype and both their mother and offspring’s latent genotype is set to 0.5. The *β_m_* and *β_o_* path coefficients refer to maternal and offspring genetic effects on birthweight, respectively, and are equivalent to the coefficients estimated using the conditional linear model in Equation (1). The residual error terms for the birthweight of the individual and their offspring are represented by *ɛ* and *ɛ_O_*, respectively, and the variance of both these terms is estimated in the SEM. The covariance between the error terms is denoted by *ρ*. Models can be fit by maximum likelihood using the OpenMx software package.^10^ We have included an example R code that implements this SEM in the Supplementary Materials, available as Supplementary data at *IJE* online, of this manuscript.

Our approach is flexible in that the results from ‘singletons’ can be incorporated into the analysis (i.e. genotyped individuals who have either their own or their offspring’s birthweight available) and the results from the SEM can be meta-analysed with results from conditional genetic association analyses of genotyped mother–offspring pairs. Importantly, our method can incorporate both individual-level genotype data (e.g. from the UK Biobank Study) and summary-level GWAS results data from other research groups and publicly available websites. The result is an extremely large, powerful, combined dataset for the estimation of maternal and offspring effects on birthweight. Additionally, when the model is applied across the genome (e.g. for locus-detection purposes), it can increase the power to detect variants that have opposing maternal and offspring effects on birthweight that may not otherwise be apparent.^4^

**Figure 2.**
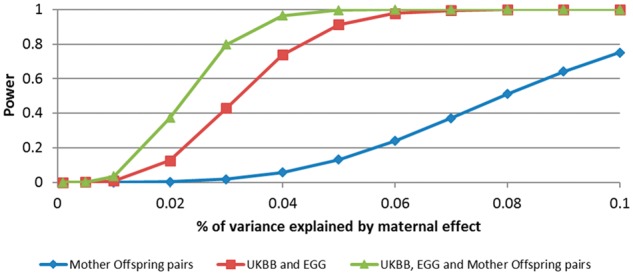
Power to detect maternal effects on birthweight as a function of variance explained. We assume a residual correlation of 0.2 between own birthweight and offspring birthweight and that the same locus exerts a maternal effect only. We compare power for *N* = 50 000 genotyped mother–offspring pairs (i.e. which is an estimate of the current number of available genotyped mother–offspring pairs worldwide that could be conceivably used for these analyses) with the current number of individuals contributing to the UK Biobank and Early Growth Genetics Consortium Analysis of birthweight (i.e. number of genotyped individuals who have data on their own birthweight and their offspring’s birthweight *N* = 85 518; number of genotyped individuals who have data on their own birthweight only *N* = 178 980; number of genotyped individuals who have data on their offspring’s birthweight only *N* = 93 842) at genome-wide significance (α = 5 × 10^–8^). Asymptotic power calculations were performed using the ‘Maternal and Offspring Genetic Effects Power Calculator’ (Moen *et al*., 2019^11^).

We have recently described the statistical properties of our approach, demonstrated that it yields unbiased estimates of maternal and offspring genetic effects on birthweight, gives similar answers to conditional genetic association analyses of genotyped mother–offspring pairs and has low sensitivity to random measurement error.^4^ Additionally, asymptotic power calculations using the ‘Maternal and Offspring Genetic Effects Power Calculator’^11^ (http://evansgroup.di.uq.edu.au/MGPC/) indicate that, whilst our approach is less powerful than similar-sized samples of genotyped mother–offspring pairs, the two study designs have similar power when the residual correlation between maternal and offspring phenotypes (*ρ* in Figure 1) approaches 0.5.^11^Figure 2 compares the asymptotic power to detect maternal effects on birthweight (assuming a residual correlation between maternal and offspring phenotypes of 0.2) using three different study designs. In the first study design, 50 000 genotyped mother–offspring pairs are used in a SEM asymptotically equivalent to a conditional linear model. Through our contacts within the Early Growth Genetics (EGG) consortium, we have estimated that 50 000 mother–offspring pairs with genome-wide SNP data would currently be available worldwide for meta-analysis. In the second study design, we use the SEM described in Figure 1 with the number of unrelated individuals currently available through either the UK Biobank Study or EGG consortium who have data on both their own and their offspring’s birthweight (*N* = 85 518), their own birthweight only (*N* = 178 980) or their offspring’s birthweight only (*N* = 93 842). In the third design, we combine the previous two data sources and conduct the SEM described in Figure 1. Figure 2 illustrates the advantage in power obtained by using the large combined sample from the UK Biobank Study and the EGG consortium, including individuals with both maternal and offspring phenotypes and singleton individuals with only one phenotype, compared with the power obtained from our estimate of the current number of genotyped mother–offspring pairs that are available across the world. However, the power can be increased dramatically by including all individuals together in the analysis.

**Figure 3.**
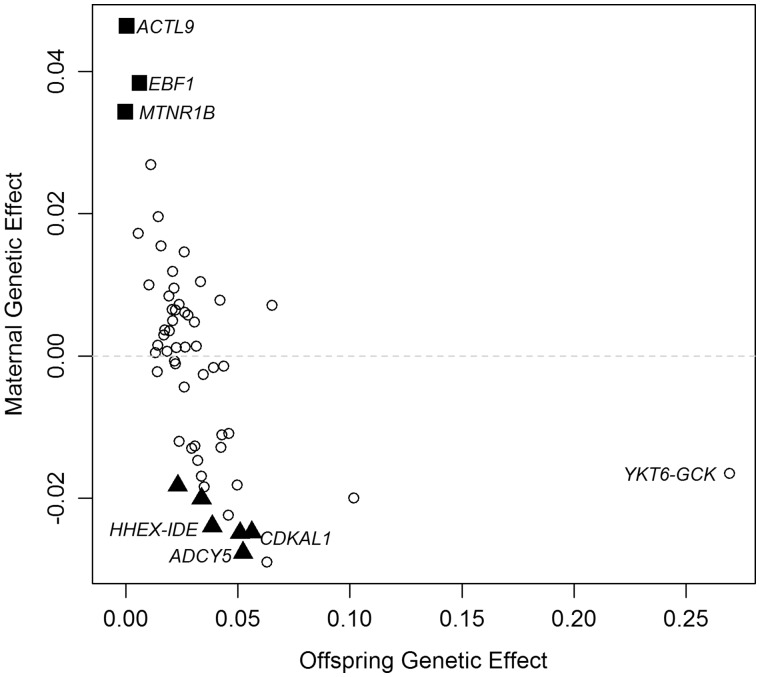
Estimated maternal and offspring genetic effects on birthweight for 58 autosomal SNPs robustly associated with birthweight. Squares highlight the subset of SNPs that exert their effects predominantly through the mother’s genome (*P*_maternal_ < 0.001 and *P*_offspring_ > 0.5). Triangles highlight the subset of SNPs with both maternal and offspring genetic effects operating in opposite directions (*P*_maternal_ < 0.05 and *P*_offspring_ < 0.05); the SNPs with their gene names are those previously associated with type 2 diabetes. The figure is based on data presented in Warrington *et al*. (2018).^4^

To further illustrate the utility of our method, we applied our SEM to 58 known autosomal SNPs for birthweight using data from the interim release of the UK Biobank Study.^8^Figure 3 displays the estimated maternal (*y*-axis) and offspring (*x*-axis) genetic effects on birthweight at each of these loci. Figure 3 shows that most loci influence birthweight primarily through the offspring genome, which was anticipated, as the loci were initially identified in a GWAS of the individual’s own birthweight. However, there is a subset of SNPs that exert their effects predominantly through the mother’s genome (e.g. *MTNR1B*, *EBF1*, *ACTL9*) and at least six loci that exhibit maternal and offspring effects in opposite directions. Interestingly, the loci that manifest opposing effects through maternal and offspring genomes include the type 2 diabetes-associated loci *HHEX-IDE*, *CDKAL1* and *ADCY5*. Our results are consistent not only with smaller and less powerful conditional regressions in genotyped mother–offspring pairs,^4^^,^^8^ but also with examples of opposing maternal and offspring contributions on the birthweight of rare mutations influencing insulin secretion and glucose tolerance, upon which the Fetal Insulin Hypothesis was based, such as those in the *GCK* gene.^12^

Whilst we have discussed our method within the context of offspring birthweight, there is no reason why the same or similar methods could not be applied to understand the genetic basis of other offspring phenotypes. Many offspring traits are thought to be influenced by maternal phenotypes^13^ and, indeed, maternal effects at specific genetic loci have been demonstrated in the case of offspring birthweight,^5^ offspring gestational age^14^ and offspring atopic dermatitis,^15^ amongst others. However, a major practical challenge in detecting/demonstrating maternal genetic effects is the paucity of large-scale cohorts with genome-wide maternal genotypes and offspring phenotypes. One way of facilitating the discovery of maternal genetics effects would be for researchers to publish complete summary GWAS results of maternal genotypes and offspring phenotypes, conditional upon offspring genotype, on publicly available websites. However, performing conditional association analyses across the genome is computationally intensive and may be difficult for some researchers to implement. A simpler alternative might be for investigators to deposit unconditional GWAS meta-analyses documenting the association between maternal genotypes and offspring phenotypes. These summary results could then be combined with GWAS of offspring genotype and phenotype in a SEM to generate unbiased estimates of maternal and offspring genetic effects on offspring phenotypes—even when the degree of sample overlap is unknown. Given the phenotypic correlation between maternal and offspring phenotypes, appropriate standard errors could then be obtained in these models by estimating the degree of sample overlap using bivariate linkage disequilibrium (LD) score regression^16^ and weighting the SEM likelihood appropriately.

**Figure 4.**
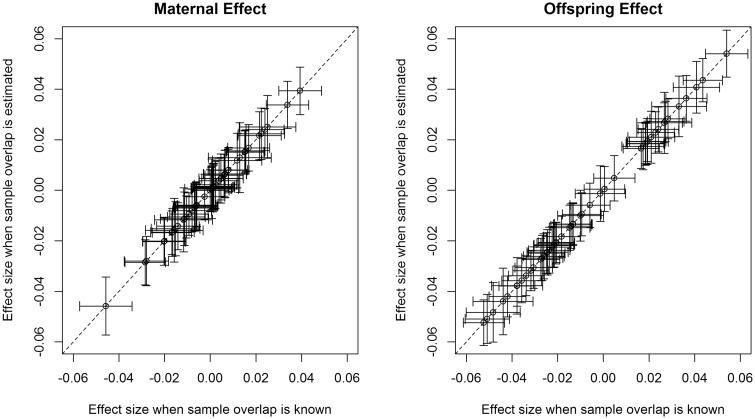
Effect sizes and standard errors for 51 autosomal birthweight-associated SNPs, which have a minor allele frequency greater than 1%, estimated from a structural equation model using covariance matrices derived from GWAS summary results of own birthweight and offspring birthweight from the UK Biobank Study. Both GWASs used z-scores of birthweight in a subset of unrelated Europeans, after adjusting for ancestry informative principal components and sex for the individual’s own birthweight (sex was not available for the birthweight of the first child in the UK Biobank Study). The *x*-axis presents results when the sample overlap is known and the *y*-axis presents results when the sample overlap is estimated using bivariate LD score regression. The phenotypic correlation between own birthweight and offspring birthweight was assumed to be 0.23 (misspecifying this correlation by small amounts i.e. *ρ *= 0.1–0.3 did not appear to influence estimates nor their standard errors for these data—results not shown).

In order to illustrate this potential, Figure 4 compares estimates of maternal (and offspring) genetic effects on birthweight at 51 known autosomal birthweight loci (with minor allele frequency greater than 1%) using summary-results GWAS data from the UK Biobank Study at various degrees of sample overlap (i.e. the same individual contributes to both the maternal and offspring GWAS summary-results data on birthweight). The column axis displays estimates (and their standard errors) from summary-results data when the degree of sample overlap is known. The second column displays the same information from summary-results data when the degree of sample overlap is unknown and has to be estimated using bivariate LD score regression.^16^ Provided the phenotypic correlation between maternal and offspring birthweight is specified relatively accurately (see Figure 4), estimates of maternal and offspring SNP effects and their standard errors are the same across the two approaches. It is likely that similar results could also be obtained using other conditional analysis strategies.^17^^,^^18^

## Using two-sample MR studies to analyse the causal effect of maternal environmental exposures on offspring phenotypes

Once genetic effects on offspring phenotypes have been partitioned into maternal and offspring components, the resulting estimates can be utilized in a variety of informative downstream analyses. In this section, we argue that the availability of maternal effect estimates on offspring phenotypes allows us to estimate the causal effect of maternal environmental exposures on offspring outcomes via two-sample MR^19^ in potentially large samples of individuals.

MR is an epidemiological method that uses genetic variants robustly associated with a modifiable environmental exposure of interest to estimate the causal relationship between the exposure and a trait or disease of interest.^1^ In the context of this manuscript, the relevant exposures are maternal traits and exposures that might affect offspring outcomes (especially whilst the offspring is *in utero*, but the method is also relevant for evaluating post-natal maternal influences on offspring phenotypes). Mendel’s Law of Segregation ensures that genetic variants segregate randomly and independently of environmental factors, whilst Mendel’s Law of Independent Assortment suggests that the genetic variants should also segregate independently of other traits, provided certain conditions are met. This means that genetic variants are less susceptible to confounding than the ‘traditional’ variables used in observational epidemiological studies.^20^ In other words, genetic variants can be used to divide a study sample into subgroups, which differ systematically with respect to the exposure of interest, but not with respect to confounding factors. If groups defined by their genotypes also show differences in their outcome, then, provided core assumptions are met,^21^ this provides evidence that the exposure causally influences the outcome. The basic MR framework has been extended successfully to two-sample situations where the SNP–exposure association is estimated in one sample and the SNP–outcome association is measured in another.^19^ This is important because it means that very precise estimates of the causal effect can be obtained by utilizing publicly available GWAS summary-results data, often using tens or hundreds of thousands of individuals.^22^

**Figure 5.**
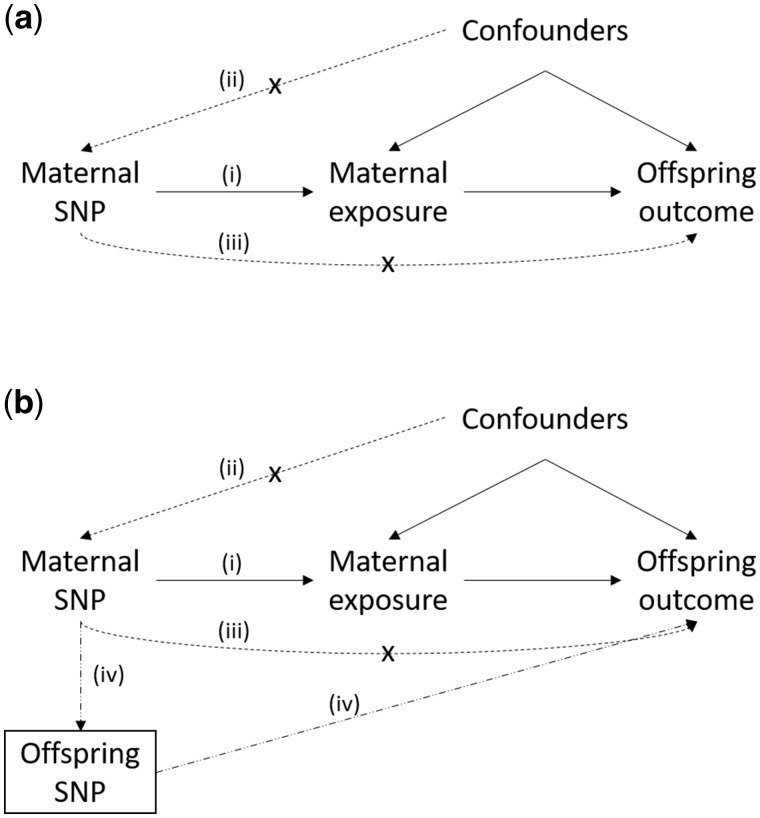
Directed acyclic graphs illustrating the core assumptions underlying Mendelian randomization. Assumption (i) requires robust association between the genetic variants and the maternal exposure. Assumption (ii) requires that the genetic variants are uncorrelated with confounders. Assumption (iii) assumes that the genetic variants are only potentially associated with the offspring outcome through the maternal exposure of interest. Offspring genetic variants violate assumption (iii), as they allow a path to offspring outcome that is not through the maternal exposure (iv). However, conditioning on offspring variants (indicated by a box around offspring SNPs) blocks path (iv) and assumption (iii) holds.

Although MR is a useful method for assessing causality, there are a number of assumptions and potential complications that should be borne in mind that we have discussed at length elsewhere.^7^^,^^23^^,^^24^ First, MR requires robust association between genetic variants and the exposure of interest [assumption (i) in Figure 5A]. Second, MR assumes that the genetic variants used are uncorrelated with confounders of the exposure–outcome relationship [assumption (ii) in Figure 5A]. Mendel’s Laws of Segregation and Independent Assortment provide assurance that this assumption is likely to be valid and there is considerable empirical evidence that this is indeed the case for many genetic variants.^20^ Third, MR assumes that the genetic instrument is only potentially associated with the outcome through the exposure of interest [assumption (iii) in Figure 5A]. This assumption, also known as the exclusion restriction criterion, precludes SNPs (or variants in LD with them) that influence multiple phenotypic traits including the exposure of interest through horizontal pleiotropy and also have an association with the outcome that is not mediated through the exposure of interest. It is widely regarded that this final assumption is likely to be more problematic for the validity of MR than the two previous assumptions, although a number of different procedures are available to detect and correct for horizontal pleiotropy.^24–29^

MR has recently been used to examine the causal effect of maternal environmental exposures on offspring outcomes. Examples include the effect of maternal cardiometabolic phenotypes^2^ and smoking^3^ on offspring birthweight and maternal alcohol consumption during pregnancy on offspring education.^30^ MR studies that attempt to estimate the causal effect of maternal exposures on offspring outcomes are subject to all the usual limitations of MR studies. However, one complication specific to estimating the causal effect of maternal environmental exposures on offspring outcomes via MR is the fact that maternal and offspring genotypes are correlated. Consequently, any association between maternal genotype and offspring outcome may in fact be mediated by offspring genotype (Figure 5B). One solution to this problem is to statistically correct for offspring’s genotype by conditioning on offspring genotype in the MR analysis.^2^^,^^7^ For example, Tyrrell *et al*.^2^ have used MR in mother–offspring pairs to show that higher maternal systolic blood pressure is likely to be causal for low offspring birthweight after conditioning on offspring genotype. However, there is a dearth of cohorts that have genotyped mother–offspring pairs, meaning that such analyses are likely to be underpowered.

One potential way to increase the power of MR analyses involving maternal exposures and offspring outcomes is to utilize two-sample MR in potentially extremely large samples of individuals.^7^^,^^19^ First, unbiased estimates of maternal genetic effects on the offspring phenotype could be obtained, e.g. from the SEM in Figure 1. These effects represent the influence of maternal genotype on offspring outcome with the effect of the offspring’s genotype removed. These estimates could then be combined with SNP–exposure estimates for the maternal exposures that the investigator is interested in, in a two-sample MR framework. In the case of offspring birthweight, the attractiveness of this strategy lies in the fact that the entire UK Biobank Study sample and data from the EGG consortium (and indeed any other publicly available summary-results statistics and large-scale cohorts) could be leveraged to estimate the association between maternal genotypes and offspring birthweight. Simultaneously, the largest publicly available GWAS meta-analysis could be used to estimate the association between the same SNPs and the maternal exposures of interest. The robustness of causal estimates, including to the possibility of latent horizontal pleiotropy, can subsequently be investigated using sensitivity analyses including MR Egger regression,^25^ weighted median MR,^26^ the MR modal estimator,^28^ multivariable MR,^29^ random-effects meta-analyses^24^ and tests of heterogeneity between the causal-effect estimates derived from different SNPs.^27^

The two-sample MR procedure we advocate relies on the assumption that the variants associated with the maternal exposure of interest are also associated with these same traits during the critical period of interest (e.g. during pregnancy) and also that it is this time point that is relevant in terms of influencing the offspring phenotype. The same assumptions are of course also made in one-sample MR studies using genotyped mother–offspring pairs—the critical difference being that, in the latter case, it may be possible to investigate these assumptions empirically.^7^ For example, there is preliminary evidence to suggest that genetic variants associated with cardiometabolic phenotypes in the general population (i.e. men and women who are not pregnant) are also associated with the same traits in mothers during pregnancy, meaning that SNPs that proxy these traits also proxy the exposures in pregnant women.^2^^,^^31^

In all the MR analyses we describe above, we assume that maternal genotypes act additively on the maternal exposure of interest; the absence of parent of origin effects (e.g. from genetic imprinting); genetic non-additivity (including genetic dominance and epistasis); no gene–gene interaction between maternal, paternal and/or offspring genotypes; and the absence of paternal genetic effects affecting the offspring outcome at the same loci used in the maternal–offspring analyses. Recent work from the deCODE group and others^13^^,^^32^ has shown that offspring phenotypes are influenced by untransmitted alleles from both parents. The presence of paternal genetic effects at the same loci used in the analysis will bias estimates of the maternal and offspring genetic effects on the offspring outcome (and consequently estimates of the causal effect of the maternal and offspring exposures on offspring outcome). This is probably more likely to be a problem where the outcome phenotype is a more ‘visible’ lifestyle characteristic, such as smoking, alcohol consumption, diet or physical activity, which could subsequently influence the offspring or that the offspring might mimic or be encouraged to adopt, than it is for something like a circulating biomarker, such as fasting glucose or vitamin D. In order to account for the effect of paternal genetic effects, paternal genotypes at the same loci would need to be included in the statistical model. Whilst this could be accomplished relatively easily in the structural equation modelling framework that we have espoused in this manuscript, doing so might be difficult in practice because of the small numbers of cohorts that have obtained genome-wide paternal genotype data on their participants. We have summarized some of the potential issues/limitations specific to MR studies of maternal exposures and offspring outcomes in Table 1.

**Table 1.** Potential limitations of MR studies of maternal exposures and offspring outcomes and suggestions of how to deal with them. We do not list limitations that are endemic to all types of MR studies, but rather focus on issues that are specific to MR studies of maternal exposures and offspring outcomes

Potential limitation	Description	Solution
Suitability of genetic variants to proxy maternal environmental exposure of interest	A key question is whether genetic variants identified in GWAS of men and (non-pregnant) women are appropriate instruments for the research question, e.g. if the interest is on the effect of maternal environmental exposures during pregnancy on offspring outcomes, is it appropriate to use SNP effects from a GWAS of the environmental exposure in another population?	Where possible utilize estimates of the association of SNPs with maternal exposures in population of interest during time period of interest
Timing of maternal exposure	Since an individual’s genetic variants are present from conception, causal estimates derived from MR studies are often thought to represent life-long effects of the environmental exposure. Interpretation of these estimates may be difficult if the investigator is interested in the effect of the maternal exposure during a particular time period (e.g. prenatal exposures)	See above.
		Examining the causal effect of paternal exposures on offspring outcomes may be informative. Evidence for similar maternal and paternal effects on offspring outcomes is consistent with post-natal effects of the environmental exposure, whereas evidence for maternal-specific effects on offspring outcomes in the absence of (or considerably weaker) paternal effects is more consistent with prenatal effects of the environmental exposure, although maternal-specific effects for some exposures may reflect a stronger postnatal maternal effect
Paternal genetic effects	Paternal genotypes at the same (or correlated) SNPs may have effects on the study exposure/outcome. Failure to take these effects into account may result in biased estimates of the causal effect of the maternal exposure on the offspring outcome	Include paternal genotypes in the statistical model where possible
Low power	MR studies may have low power because individual SNPs explain small portions of variance in the exposure and the outcome. This potential limitation may be exacerbated in MR studies of maternal exposures because the causal effect of the maternal exposure on the offspring outcome may be smaller than the effect of the maternal exposure on maternal outcomes (as is examined in typical MR studies)	Utilize multiple instruments that explain more variance in the maternal exposure
		Utilize two-sample MR methods described in this manuscript to increase sample size and statistical power
Violation of exclusion restriction criteria via offspring genetic effects on offspring outcome	Maternal SNPs may be associated with offspring outcome via their association with offspring genotype violating the exclusion restriction assumption of MR and biasing causal estimates	Perform MR analyses conditioning on offspring genotype
		Utilize two-sample MR methods described in this manuscript
Paucity of genotyped mother–offspring pairs	There is a dearth of cohorts worldwide that contain large numbers of genotyped mother–offspring pairs for MR analyses of maternal exposures meaning that these sorts of analyses may lack power	Utilize two-sample MR methods described in this manuscript to combine summary-results information across many different cohorts

Finally, we note that an alternative approach consisting of fitting a multivariate model that includes maternal genotype, offspring genotype, maternal exposure and offspring outcome (e.g. via multivariable MR^29^) would likely yield similar causal estimates of the maternal exposure on the offspring outcome to the framework that we espouse in this manuscript. However, we emphasize that, regardless of which approach is used, the important point is that both methods are capable of generating and utilizing estimates of the conditional effect of maternal genotype on offspring phenotype in two-sample data, increasing the number of individuals contributing to the analysis and statistical power to detect the causal effect of maternal exposures on offspring outcomes.

## Using maternal genetic effects to investigate the effect of exposures influencing fetal growth on future offspring health and disease

One exposure that is of interest to the epidemiological community is fetal growth and the *in utero* exposures that influence it, and their effect on future offspring outcomes. DOHaD posits that adverse intrauterine environments result in different fetal growth trajectories (some resulting in growth restriction, others overgrowth) and (independently) increased future risk of disease through developmental changes.^6^ However, the *in utero* environment is difficult to measure and investigators may settle with infant birthweight as a proxy of this. There are several problems with this methodology, including the fact that offspring birthweight is an imperfect measure of growth *in utero* as well as all the usual limitations of traditional observational epidemiological studies (confounding, etc.). We believe that SNPs in mothers that are robustly associated with offspring birthweight (i.e. after conditioning on offspring genotype) exert their effects through the *in utero* environment and consequently that these variants could be used to proxy intrauterine growth restriction. Therefore, it should be possible to use the principles of MR to investigate the causal effect of *in utero* exposures influencing fetal growth on a variety of offspring phenotypes. Specifically, we propose that the existence of birthweight-associated SNPs in the mother that also exert maternal genetic effects on other offspring outcomes is highly suggestive of mechanisms consistent with DOHaD.

**Figure 6.**
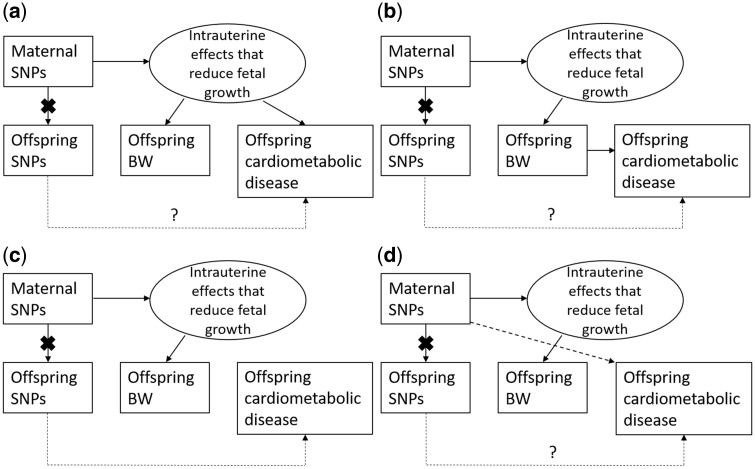
This figure illustrates the four possible ways in which maternal SNPs that are associated with offspring birthweight (conditional on offspring genotype at the same locus) can also be (unconditionally) associated with offspring cardiometabolic disease risk. The ‘X’ represents the effect of conditioning the association analysis on either the offspring or maternal genotype and therefore blocking the path between the conditioned genotype and the other variables of interest. The dashed path with the question mark indicates the potential pleiotropic effects of the offspring’s SNPs on their own cardiometabolic disease risk.

To illustrate why this is the case, Figure 6 shows the four credible ways in which SNPs in the mother that exert maternal effects on offspring birthweight can also be associated with future offspring phenotypes (although we have used cardiometabolic disease in this example, similar diagrams could be used to illustrate the relationship between *in utero* exposures influencing fetal growth and other offspring outcomes). In the different panels of this figure, we consider the relationship between SNPs in the mother that are associated with offspring birthweight (i.e. through maternal genetic effects) and cardiometabolic disease in the offspring. The black ‘X’ in the diagram represents the effect of conditioning the association analysis on either the offspring or maternal genotype and therefore blocking the path between the conditioned genotype and the other variables of interest.

In panel (A), and consistently with DOHaD, SNPs in mothers that produce an adverse *in utero* environment lead to reduced fetal growth and subsequently lower offspring birthweight and developmental compensations that result in increased risk of offspring cardiometabolic disease in later life. Under this scenario, SNPs in the maternal genome that exert maternal effects to reduce offspring birthweight will also be positively correlated with offspring cardiometabolic risk (after correcting for offspring genotype). In panel (B), lower offspring birthweight is causal for increased risk of cardiometabolic disease. Under this scenario, SNPs in the maternal genome that exert maternal effects to reduce offspring birthweight will also be positively associated with offspring cardiometabolic disease risk (after correcting for offspring genotype). We stress that, although this model is broadly consistent with DOHaD, most advocates of DOHaD would not believe that birthweight is directly causal for future cardiometabolic risk, but rather a marker of an adverse *in utero* environment, as in panel (A). Nevertheless, we believe it is important to consider this possibility, particularly as naïve MR analyses making similar assumptions have begun to appear in the literature.^33^ Importantly, under panels (A) and (B), the existence of a negative association between maternal SNPs that are associated with lower offspring birthweight and increases in offspring cardiometabolic phenotypes is consistent with DOHaD.

In panel (C), the correlation between SNPs in the mother and offspring cardiometabolic risk is driven by genetic pleiotropy in the offspring genome. Under this model, SNPs that exert maternal effects on offspring birthweight will not be correlated with offspring cardiometabolic risk after conditioning on offspring genotype. This model encompasses the possibility of cardiometabolic disease-associated SNPs in mothers directly affecting offspring birthweight and then being transmitted to their offspring, where they increase the risk of cardiometabolic disease in later life.

Finally, in panel (D), SNPs that exert maternal effects on offspring birthweight also pleiotropically influence offspring cardiometabolic risk through the postnatal environment. Under this model, SNPs with maternal effects on birthweight will be associated with offspring cardiometabolic risk after conditioning on offspring genotype. In general, however, we think this last model is unlikely, since the primary effect of these variants is likely to be on birthweight though the *in utero* environment. Any postnatal environmental effects of these variants, if they exist, on offspring phenotypes are likely to be small in comparison to their *in utero* effects. We note that the availability of mature genotyped father–offspring pairs would provide a test of this assumption, since we would expect that paternal genotypes will not be associated with offspring cardiometabolic risk in the absence of postnatal environmental effects.

Thus, the different models illustrated in Figure 6 imply different patterns of association between maternal and offspring SNPs, offspring birthweight and offspring cardiometabolic outcomes according to the source, nature and direction of the underlying relationships. In particular, the existence of negative associations between maternal birthweight-associated SNPs and offspring cardiometabolic outcomes (after conditioning on offspring genotype) strongly argues for the importance of intrauterine growth restriction in this context. We therefore posit that tests of the maternal effect of SNPs on offspring traits (whilst conditioning on offspring genotype) are akin to testing the validity of DOHaD in this particular context. We note that there exist opportunities to test other models of disease development using similar frameworks, including the Fetal Insulin Hypothesis.^12^ For example, under this hypothesis, we would predict positive relationships between maternal birthweight-associated SNPs and offspring glycaemic variables, but negative correlations between offspring birthweight-associated SNPs and offspring glycaemic variables.

In this section, we have used birthweight SNPs to proxy intrauterine growth restriction and have consequently made the assumption that variation across the distribution of birthweight is informative for inference regarding the validity of DOHaD. Whilst utilizing birthweight-associated SNPs represents a useful starting point for investigations, some DOHaD literature concerns the effect of more extreme maternal exposures (e.g. famine) on fetal growth restriction and subsequent adaptation.^34^ It may well be that these sorts of exposures produce changes that are qualitatively different from small perturbations within the normal range. However, there is no reason why the aforementioned methodologies could not be modified to examine these more extreme situations. For example, it would be interesting to investigate the effect of pre-eclampsia on birthweight and subsequent offspring cardiometabolic risk. Again, a partitioning of genetic effects of pre-eclampsia loci into maternal and offspring components would be a necessary prerequisite for understanding the processes involved.

## Applied example

In order to illustrate the framework espoused in this manuscript, we examined the relationship between maternal and offspring susceptibility to type 2 diabetes and offspring birthweight in the UK Biobank study^9^ (*N* = 234 154 individuals reporting their own birthweight and *N* = 210 423 mothers reporting their offspring’s birthweight). Full details on the sample, genotyping and phenotypes are included in the Supplementary Materials, available as Supplementary data at *IJE* online. A total of 403 SNPs identified in a recent GWAS meta-analysis of type 2 diabetes^35^ were extracted from the imputed files provided by UK Biobank and aligned to the type 2 diabetes risk allele (Supplementary Table 1, available as Supplementary data at *IJE* online). We fit the SEM described in Figure 1 to the data from each of these 403 SNPs to estimate the maternal and offspring genetic effects on birthweight. One of the 403 SNPs, rs79046683, had a low minor allele frequency in the UK Biobank (MAF = 3.9 × 10^–5^), so the SEM was unable to converge using this SNP and it was therefore excluded from subsequent MR analyses. Using the effect sizes for type 2 diabetes reported in Mahajan *et al*.,^35^ we calculated the Wald ratio estimate of the causal effect of maternal susceptibility to type 2 diabetes on offspring birthweight at each of the 402 SNPs (i.e. calculated as the SEM-derived maternal genetic effect of the SNP on offspring birthweight divided by the logistic regression coefficient of the SNP association with type 2 diabetes). Additionally, we calculated the causal effect of an individual’s own susceptibility to type 2 diabetes on their own birthweight (i.e. calculated as the SEM-derived offspring genetic effect of the SNP on offspring birthweight divided by the logistic regression coefficient of the SNP association with type 2 diabetes). Using these Wald statistics, we calculated an inverse variance-weighted (IVW) causal-effect estimate, using a random-effects model for the standard error. We also used MR Egger regression to provide estimates of the causal effect robust to violations of the exclusion restriction assumption caused by horizontal pleiotropy.^25^ Finally, in order to compare our results to those that would have been obtained had we not partitioned genetic effects into maternal and offspring components using our SEM, we generated causal-effect estimates using uncorrected estimates of the SNP–own birthweight association and uncorrected estimates of the SNP–offspring birthweight association using simple linear regression. Since these latter analyses do not explicitly partition genetic effects on birthweight into maternal and offspring genetic components, the SNP–birthweight associations will reflect a complicated mixture of maternal and offspring genetic effects and will likely bias downstream MR analyses.

The results of our analyses are displayed in Table 2. We found strong evidence for a positive causal effect of maternal susceptibility to type 2 diabetes on offspring birthweight. In contrast, type 2 diabetes alleles transmitted to offspring were associated with reduced birthweight. This pattern of results was seen using both IVW MR and MR Egger approaches and is entirely consistent with both the Developmental Overnutrition and Fetal Insulin Hypotheses models of the relationship between type 2 diabetes and birthweight.^12^ In short, mothers who have or are susceptible to type 2 diabetes are likely to have poorer glycemic control and higher blood glucose, resulting in increased fetal growth and higher offspring birthweight (Developmental Overnutrition). Conversely, children who receive type 2 diabetes susceptibility alleles from their parents are less sensitive to insulin and therefore tend to have lower birthweights on average, resulting in an inverse association of birthweight with subsequent type 2 diabetes (Fetal Insulin Hypothesis).

**Table 2.** Causal estimates of maternal (and offspring) susceptibility to type 2 diabetes on offspring birthweight using two-sample Mendelian randomization. Results are presented using unadjusted estimates (i.e. estimates of the SNP–birthweight association used in the MR analysis were not corrected for the correlation between maternal and offspring genotypes) and adjusted estimates where the maternal and offspring genetic effects on birthweight were first obtained through the SEM (i.e. estimates of the SNP–birthweight association used in the MR analysis were first corrected for the correlation between maternal and offspring genotypes using the SEM). Causal estimates are presented (*β*), their standard errors (in parentheses) and *P*-values from the analysis. Causal estimates represent the estimated difference in mean birthweight in standard deviation units comparing infants whose mothers are susceptible to type 2 diabetes to those mothers who are not (Maternal effect) and the estimated difference in mean birthweight in standard deviation units comparing infants who are themselves susceptible to type 2 diabetes vs those who are not (Offspring effect)

	Adjusted estimates (using SEM)		Unadjusted estimates	
Method	Maternal effect	Offspring effect	Maternal effect	Offspring effect
IVW MR	β = 0.036 (0.007), *P* = 1.8 × 10^–7^	β = –0.043 (0.007), *P* = 7.0 × 10^–9^	β = 0.015 (0.007), *P* = 0.022	β = –0.024 (0.007), *P* = 3.7 × 10^–3^
Egger regression	β = 0.030 (0.013), *P* = 0.021	β = –0.028 (0.014), *P* = 0.042	β = 0.016 (0.012), *P* = 0.198	β = –0.013 (0.013), *P* = 0.313

IVW MR, inverse variance-weighted Mendelian randomization.

Importantly, causal estimates obtained using the SEM-adjusted estimates of the maternal and offspring genetic effects were far greater in magnitude than causal estimates obtained using the uncorrected estimates from simple linear regression. Likewise, the strength of evidence against the null hypothesis of no association was far greater for the SEM-derived estimates. Our results suggest that failing to partition genetic effects into maternal and offspring genetic components has the potential to bias the results of MR analyses and lead to misleading interpretations of the data. R code illustrating the SEM and two-sample MR analyses we have performed are included in the Supplementary Materials, available as Supplementary data at *IJE* online.

## Future directions

We envisage that the major challenge in implementing the ideas outlined in this manuscript will be practical rather than technical. With some notable exceptions,^5^^,^^14^ most large-scale international genetics consortia have involved GWAS of individuals’ own genotype and phenotype. In general, these efforts have been extraordinarily successful and responsible for the robust identification of thousands of genetic loci associated with complex traits and diseases.^36^ Consequently, the majority of summary GWAS results data posted on publicly available websites concern these associations. We propose the establishment of analogous international consortia (or efforts within existing consortia) whose remit is the detection of maternal genetic effects on offspring phenotypes and who are committed to the deposition of summary-results data from these collaborations on publicly available servers and utilities such as MRBase^37^ and LDHub.^38^ This will not only facilitate the detection of maternal genetic effects on offspring phenotypes, but also enable large-scale MR studies of maternal exposures and offspring outcomes in the wider scientific community.

A logical place to start might be the establishment of working groups focused on perinatal phenotypes, where maternal genetic effects on offspring phenotypes are expected to be strongest. We have already begun to do this in the case of offspring birthweight within the EGG consortium, where maternal genotypes and offspring phenotypes in the large-scale UK Biobank Study and many other population-based birth cohorts of appreciable size are available.^5^ However, many other perinatal phenotypes have been collected by cohorts within both the EGG and Early Genetics and Lifecourse Epidemiology (EAGLE) consortia, which would facilitate the examination of other traits, too. There also exist several very large Scandinavian cohorts that have maternal genotypes and offspring outcomes, including the Norwegian Mother and Child Cohort (MOBA),^39^ the HUNT Study^40^^,^^41^ and DECODE^13^ cohorts, and several smaller twin and family studies cohorts and family studies with parental genotypes available that would be useful for this purpose (Table 3).

**Table 3.** List of cohorts that have maternal genotype data and offspring phenotype data

Cohort	Approximate number of genotyped mother–phenotyped child pairsa
1958 British Birth Cohort (B85C-T1DGC)^42^	858^5^
1958 British Birth Cohort (B85C-WTCCC)^42^	836^5^
Add Health—National Longitudinal Study of Adolescent to Adult Health^43^	∼1000
Autism Genome Project (AGP)^44^	2594^45^
Avon Longitudinal Study of Parents and Children (ALSPAC)^46^	7304^5^
Berlin Birth Cohort (BBC)^47^	1357^2^
Born in Bradford Study (BiB)^48^	∼10 000
Chicago Food Allergy Study^49^	541^50^
Children’s Hospital of Philadelphia (CHOP)	312^2^
Copenhagen Prospective Study on Asthma in Childhood (COPSAC-2000)^51^	282^2^
Danish National Birth Cohort—Genomics of Young Adolescent (DNBC-GOYA)^52^	1805^5^
Danish National Birth Cohort—Preterm Birth Study (DNBC-PTB)^53^	1656^5^
deCODE (Genealogy Database)^54^	54 546^13^
Environmental Risk (E-Risk) Longitudinal Twin Study^55^	804^56^
Exeter Family Study of Childhood Health (EFSOCH)^57^	746^2^
Family Atherosclerosis Monitoring In earLY life (FAMILY) study^58^	406^59^
Finnish Twin Cohort^60^	∼4000^61^
Hispanic B-cell Acute Lymphoblastic Leukemia Study^62^	323^62^
HUNT Study^41^	∼18 000
Hyperglycemia and Adverse Pregnancy Outcome Study (HAPO)^63^	4437^63^
Millennium Cohort^64^	12 000
Minnesota Center for Twin and Family Research (MCTFR)^65^	1404^65^
Netherlands Twin Register (NTR)^66^	707^5^
Northern Finland 1966 Birth Cohort Study (NFBC1966)^67^	2035^5^
Norwegian Mother and Child Cohort Study (MoBa)^39^	∼46 000
Prediction and Prevention of Preeclampsia and Intrauterine Growth Restriction Study (PREDO)^68^	∼1000
Pune Maternal Nutrition Study (PMNS)^69^	533^69^
QIMR Berghofer Cohort^70^	892^5^
Simons Simplex Collection^71^	2576^71^
Sister Study^72^	715^72^
STORK Study^73^	529^31^^,^^73^
STORK Groruddalen^74^	634
TwinsUK^75^	1603^5^
UK Biobank^b^^,^^76^	221 528

a The number of genotyped mother–phenotyped child duos is based on information provided in peer-reviewed papers including on birthweight^5^ and gestational weight gain,^77^ on the cohort’s official website or from discussions with the study principal investigators. These numbers are liable to change as more individuals are recruited/genotyped, and should only be considered approximations.

b Birthweight only.

## Conclusions

Statistical methods now exist for estimating maternal genetic effects on offspring phenotypes. The estimates obtained in these studies can subsequently be used in large-scale two-sample MR studies to investigate the causal effect of maternal environmental exposures on offspring outcomes. This includes studies that aim to assess the causal effect of *in utero* exposures influencing fetal growth restriction on future risk of disease in offspring. The establishment of large-scale international genetics consortia geared to identifying maternal genetic effects will assist in facilitating these sorts of studies.

## Funding

D.M.E. is supported by an NHMRC Senior Research Fellowship (GNT1137714) and this work was supported by project grants (GNT1157714, GNT1125200). G.H.M. has a PhD grant from the South-Eastern Norway Regional Health Authority (Grant number 2015008). D.A.L.’s contribution to this piece is supported by the European Research Council under the European Union’s Seventh Framework Programme (FP/2007–2013)/ERC Grant Agreement (Grant number 669545; DevelopObese), the European Union’s Horizon 2020 research and innovation programme under grant agreement No. 733206 (LifeCycle) and the United States National Institutes of Health (NIH): National Institute of Diabetes and Digestive and Kidney Diseases (R01 DK10324). D.M.E. and D.A.L. work in a UK Medical Research Council (MRC) Unit that is supported by the University of Bristol and MRC (MC_UU_00011/6) and D.A.L. is a National Institute of Health Research Senior Investigator (NF-SI-0611–10196). N.M.W. is supported by an Australian National Health and Medical Research Council Early Career Fellowship (GNT1104818). This research has been conducted using the UK Biobank Resource (Reference 12703). Access to the UKBB study data was funded by a University of Queensland Early Career Researcher Grant (2014002959). No funders influenced the study design, data collection or interpretation of results. The views expressed in this paper are those of the authors.

## Supplementary Material

dyz019-Supplementary_Data
