# Supplementary material for: Elucidating the role of maternal environmental exposures on offspring health and disease using two-sample Mendelian randomization
Source: Int J Epidemiol. 2019 Feb 27;48(3):861–75. doi: 10.1093/ije/dyz019 (PMC6659380; doi:10.1093/ije/dyz019)
Supplement: dyz019-Supplementary_Data [file dyz019-supplementary_data.zip › dyz019-suppl_data/Supplementary Methods.docx]

Supplementary Methods

UK Biobank phenotype data were available on 502,543 individuals, of which 280,142 reported their own birthweight at either the baseline or one of the follow-up visits. There were 7,701 individuals who were part of multiple births and were excluded from the analyses. Of the 10,958 individuals who reported their own birthweight at more than one follow-up, 83 were excluded because the two values differed by more than 1kg. For those individuals who reported different values between the follow-ups (<1kg) we took the average of the available measures the analyses. Finally, we excluded 38,204 individuals who reported their own birthweight to be <2.5kg or >4.5kg, as these are implausible for live term births before 1970. In total, 234,154 individuals had data on their own birthweight matching our inclusion criteria.

Women in the UK Biobank were also asked to report the birthweight of their first child to the nearest pound. We used the same inclusion criteria as for their own birthweight, except we excluded individuals with birthweight of their first child <2.25kg or >4.6kg (which equates to <5 pounds and >10 pounds). This left 210,423 individuals with birthweight of their first child (29 excluded because the multiple reports of offspring birthweight differed by >1kg; 6,330 excluded with birthweight of their first child <2.25kg or >4.6kg).

Genotype data from the full release (April 2018) were used for the analysis. In addition to the quality control metrics performed centrally by the UK Biobank, we excluded individuals who were related. We defined a subset of ‘white European’ ancestry samples using a *K*-means (K=4) clustering approach based on the first four genetically determined principal components. A subset of 257,696 unrelated individuals with genotype data, a valid birthweight for themselves or their first child and were genetically of ‘white European’ ancestry were included in the analysis. Of these, 72,274 were men who only reported their own birthweight. Among the women, 25,951 reported only their own birthweight, 73,968 reported only that of their first child, and 85 503 reported both. We adjusted both the individual’s own birthweight and the birthweight of their first child for the first 40 principal components to account for any remaining population substructure, assessment centre and genotyping chip and we also adjusted the individual’s own birthweight for sex (sex was not reported for the offspring). Finally, we created z-scores from the adjusted birthweight values.

Example R code for fitting the SEM illustrated in Figure 1 to the UK Biobank birthweight data and then performing the two sample MR analysis of maternal type 2 diabetes susceptibility and offspring birthweight

######################

### Open libraries ###

######################

require(OpenMx)

########################

### SEM for all SNPs ###

########################

results <- data.frame() # create an empty data frame to store results

# data is a data frame with own birth weight ('bw_z') in the first column, offspring birth weight ('bw_off_z') for the women in the second column and then a column

# for each of the SNPs of interest (code works for SNPs labelled with 'rs...')

for(i in 3:ncol(data)){

data_sub <- data[,c(1,2,i)]

manifests <- names(data_sub)

snps <- names(data_sub)[grep("rs", names(data_sub))]

gg <- paste("gg_", snps, sep="")

go <- paste("go_", snps, sep="")

mvar <- paste("mvar_", snps, sep="")

ovar <- paste("ovar_", snps, sep="")

latents <- c("e1", "e2", go, gg, mvar, ovar)

mean_lab <- paste("mean_", manifests, sep="")

snps_c_bw_lab <- paste("c_", snps, sep="")

snps_m_bw_lab <- paste("m_", snps, sep="")

theta_lab <- paste("theta_", snps, sep="")

### Model including maternal and fetal effect estimates

SEMmodel <- mxModel(model = "UKB", type="RAM", mxData(observed=data_sub, type="raw"),

mxPath(from=gg, arrows=2, free=TRUE, values=c(rep(0.2,1)), labels=theta_lab), #Variance of 'grandmother' latent genetic variables

mxPath(from=mvar, arrows=2, free=TRUE, values=c(rep(0.2,1)), labels=theta_lab), #Variance of 'mother' latent genetic variables

mxPath(from=ovar, arrows=2, free=TRUE, values=c(rep(0.2,1)), labels=theta_lab), #Variance of 'offspring' latent genetic variables

mxPath(from=mvar, to=snps, arrows=1, free=FALSE, values=sqrt(0.75), labels="MV"), #Path from maternal latent variable for variance to SNP. Value set to sqrt(0.75)

mxPath(from=ovar, to=go, arrows=1, free=FALSE, values=sqrt(0.75), labels="OV"), #Path from offspring latent variable for variance to latent genetic variable. Value set to sqrt(0.75)

mxPath(from=c("e1", "e2"), arrows=2, free=TRUE, values=1, labels=c("var_e1", "var_e2")), #Variance of residual errors

mxPath(from="e1", to="e2", arrows=2, free=TRUE, values=0.2, labels=c("phi1")), #Correlation between residual errors

mxPath(from="e1", to="bw_z", arrows=1, free=FALSE, values=1, labels=c("e1")), #Residual error of maternal birthweight. Value set to 1.

mxPath(from="e2", to="bw_off_z", arrows=1, free=FALSE, values=1, labels=c("e3")), #Residual error offspring birthweight. Value set to 1.

mxPath(from=snps, to="bw_z", arrows=1, free=TRUE, values=0, labels=snps_c_bw_lab), #Effect of SNP on maternal BW (fetal effect)

mxPath(from=snps, to="bw_off_z", arrows=1, free=TRUE, values=0, labels=snps_m_bw_lab), #Effect of SNP on offspring BW (maternal effect)

mxPath(from=gg, to="bw_z", arrows=1, free=TRUE, values=0, labels=snps_m_bw_lab), #Effect of grandmothers genes on maternal BW (maternal effect)

mxPath(from=go, to="bw_off_z", arrows=1, free=TRUE, values=0, labels=snps_c_bw_lab), #Effect of offsprings genes on offspring BW (fetal effect)

mxPath(from=gg, to=snps, arrows=1, free=FALSE, values=0.5, labels="G"), #Path from grandmothers genes to maternal SNP. Value set to 0.5

mxPath(from=snps, to=go, arrows=1, free=FALSE, values=0.5, labels="O"), #Path from maternal SNP to offspring genes. Value set to 0.5

mxPath( from="one", to=manifests, arrows=1, free=TRUE, values=rep(0,length(manifests)), labels=mean_lab), # means and intercepts

manifestVars = manifests, latentVars = latents

)

# run model

SEMfit <- mxTryHard(SEMmodel, extraTries=1000)

Zscore <- summary(SEMfit)$parameters[,5]/summary(SEMfit)$parameters[,6]

Pval <- ifelse(Zscore<0, 2*(1-pnorm(abs(Zscore))), 2*(pnorm(abs(Zscore), lower.tail=FALSE)))

test <- cbind(Zscore, Pval)

### Model without maternal and fetal effects to calculate 2DF test

SEMmodel_base <- mxModel(model = "UKB_base", type="RAM", mxData(observed=data_sub, type="raw"),

mxPath(from=gg, arrows=2, free=TRUE, values=c(rep(0.2,1)), labels=theta_lab), #Variance of 'grandmother' latent genetic variables

mxPath(from=mvar, arrows=2, free=TRUE, values=c(rep(0.2,1)), labels=theta_lab), #Variance of 'mother' latent genetic variables

mxPath(from=ovar, arrows=2, free=TRUE, values=c(rep(0.2,1)), labels=theta_lab), #Variance of 'offspring' latent genetic variables

mxPath(from=mvar, to=snps, arrows=1, free=FALSE, values=sqrt(0.75), labels="MV"), #Path from maternal latent variable for variance to SNP. Value set to sqrt(0.75)

mxPath(from=ovar, to=go, arrows=1, free=FALSE, values=sqrt(0.75), labels="OV"), #Path from offspring latent variable for variance to latent genetic variable. Value set to sqrt(0.75)

mxPath(from=c("e1", "e2"), arrows=2, free=TRUE, values=1, labels=c("var_e1", "var_e2")), #Variance of residual errors

mxPath(from="e1", to="e2", arrows=2, free=TRUE, values=0.2, labels=c("phi1")), #Correlation between residual errors

mxPath(from="e1", to="bw_z", arrows=1, free=FALSE, values=1, labels=c("e1")), #Residual error of maternal birthweight. Value set to 1.

mxPath(from="e2", to="bw_off_z", arrows=1, free=FALSE, values=1, labels=c("e3")), #Residual error offspring birthweight. Value set to 1.

mxPath(from=gg, to=snps, arrows=1, free=FALSE, values=0.5, labels="G"), #Path from grandmothers genes to maternal SNP. Value set to 0.5

mxPath(from=snps, to=go, arrows=1, free=FALSE, values=0.5, labels="O"), #Path from maternal SNPs to offspring genes. Value set to 0.5

mxPath( from="one", to=manifests, arrows=1, free=TRUE, values=rep(0,length(manifests)), labels=mean_lab), # means and intercepts

manifestVars = manifests, latentVars = latents

)

# run model

SEMfit_base <- mxTryHard(SEMmodel_base, extraTries=1000)

### Linear models for comparison

lm_c <- lm(data_sub$bw_z ~ data_sub[,3])

lm_m <- lm(data_sub$bw_off_z ~ data_sub[,3])

if(SEMfit$output$status$code==0 && SEMfit_base$output$status$code==0){

results <- rbind(results, c(summary(SEMfit)$parameters[1,5], summary(SEMfit)$parameters[1,6], test[1,1], test[1,2],

summary(SEMfit)$parameters[2,5], summary(SEMfit)$parameters[2,6], test[2,1], test[2,2], mxCompare(SEMfit, SEMfit_base)[2,9],

summary(lm_c)$coefficients[2,1], summary(lm_c)$coefficients[2,2], summary(lm_c)$coefficients[2,4],

summary(lm_m)$coefficients[2,1], summary(lm_m)$coefficients[2,2], summary(lm_m)$coefficients[2,4], 0))

}

else{

results <- rbind(results, c(summary(SEMfit)$parameters[1,5], summary(SEMfit)$parameters[1,6], test[1,1], test[1,2],

summary(SEMfit)$parameters[2,5], summary(SEMfit)$parameters[2,6], test[2,1], test[2,2], mxCompare(SEMfit, SEMfit_base)[2,9],

summary(lm_c)$coefficients[2,1], summary(lm_c)$coefficients[2,2], summary(lm_c)$coefficients[2,4],

summary(lm_m)$coefficients[2,1], summary(lm_m)$coefficients[2,2], summary(lm_m)$coefficients[2,4], 1))

}

}

results <- cbind(names(data_sub_bw)[1:(length(names(data_sub_bw))-2)], results)

names(results) <- c("SNP", "SEM_Beta_c", "SEM_SE_c", "SEM_Z_c", "SEM_P_c", "SEM_Beta_m", "SEM_SE_m", "SEM_Z_m", "SEM_P_m", "SEM_2DF_P", "LM_Beta_c", "LM_SE_c", "LM_P_c",

"LM_Beta_m", "LM_SE_m", "LM_P_m", "ModelStatus")

##########################################

### Merge T2D summary stats to results ###

##########################################

# Download the summary statistics from supplementary table 2 in the following paper:

# Mahajan A, Taliun D, Thurner M, Robertson NR, Torres JM, Rayner NW, et al. Fine-mapping type 2 diabetes loci to single-variant resolution using high-density imputation

# and islet-specific epigenome maps. Nat Genet. 2018;50(11):1505-13. (https://www.nature.com/articles/s41588-018-0241-6)

# Format it so that the odds ratio (OR) and 95% confidence intervals (95% CI) are in separate columns and then load it into a data frame called 'details'

results <- merge(details, results, by.x="Index.variant", by.y="SNP", all.y=T)

dim(results)

results$ModelStatus <- factor(results$ModelStatus, labels=c("Okay", "Caution")) #relabel the 'ModelStatus' factor to show whether the SEM fit without error ('okay') or not ('Caution')

#########################

### Write out results ###

#########################

write.table(results, "SEM_results.txt", sep="\t", row.names=F, quote=F)

###################

### MR Analysis ###

###################

### IVW using maternal and fetal effects on birth weight from SEM results

summary(lm(results$SEM_Beta_m ~ -1 + log(results$OR), weights = (1 / (results$SEM_SE_m)^2))) # maternal

summary(lm(results$SEM_Beta_c ~ -1 + log(results$OR), weights = (1 / (results$SEM_SE_c)^2))) # fetal

### MR-Egger using maternal and fetal effects on birth weight from SEM results

summary(lm(results$SEM_Beta_m ~ log(results$OR), weights = (1 / (results$SEM_SE_m)^2))) # maternal

min(summary(lm(results$SEM_Beta_m ~ log(results$OR), weights = (1 / (results$SEM_SE_m)^2)))$sigma, 1)

summary(lm(results$SEM_Beta_c ~ log(results$OR), weights = (1 / (results$SEM_SE_c)^2))) # fetal

min(summary(lm(results$SEM_Beta_c ~ log(results$OR), weights = (1 / (results$SEM_SE_c)^2)))$sigma, 1)

### IVW using maternal and fetal effects on birth weight from standard linear model

summary(lm(results$LM_Beta_m ~ -1 + log(results$OR), weights = (1 / (results$LM_SE_m)^2))) # maternal

summary(lm(results$LM_Beta_c ~ -1 + log(results$OR), weights = (1 / (results$LM_SE_c)^2))) # fetal

### MR-Eggar using maternal and fetal effects on birth weight from standard linear model

summary(lm(results$LM_Beta_m ~ log(results$OR), weights = (1 / (results$LM_SE_m)^2))) # maternal

min(summary(lm(results$LM_Beta_m ~ log(results$OR), weights = (1 / (results$LM_SE_m)^2)))$sigma, 1)

summary(lm(results$LM_Beta_c ~ log(results$OR), weights = (1 / (results$LM_SE_c)^2))) # fetal

min(summary(lm(results$LM_Beta_c ~ log(results$OR), weights = (1 / (results$LM_SE_c)^2)))$sigma, 1)
